# Considerations about the Determination of the Depolarization Calibration Profile of a Two-Telescope Lidar and Its Implications for Volume Depolarization Ratio Retrieval

**DOI:** 10.3390/s18061807

**Published:** 2018-06-04

**Authors:** Adolfo Comerón, Alejandro Rodríguez-Gómez, Michaël Sicard, Rubén Barragán, Constantino Muñoz-Porcar, Francesc Rocadenbosch, María José Granados-Muñoz

**Affiliations:** 1 CommSensLab, Department of Signal Theory and Communications, Universitat Politècnica de Catalunya (BarcelonaTech-UPC), 08034 Barcelona, Spain; comeron@tsc.upc.edu (A.C.); msicard@tsc.upc.edu (M.S.); ruben.barragan@tsc.upc.edu (R.B.); constan@tsc.upc.edu (C.M.-P.); roca@tsc.upc.edu (F.R.); maria.jose.granados@tsc.upc.edu (M.J.G.-M.); 2 Ciències i Tecnologies de l’Espai—Centre de Recerca de l’Aeronàutica i de l’Espai/Institut d’Estudis Espacials de Catalunya (CTE-CRAE/IEEC), Universitat Politècnica de Catalunya, 08034 Barcelona, Spain

**Keywords:** lidar system, depolarization channel, calibration, error compensation, depolarizing particles

## Abstract

We propose a new method for calculating the volume depolarization ratio of light backscattered by the atmosphere and a lidar system that employs an auxiliary telescope to detect the depolarized component. It takes into account the possible error in the positioning of the polarizer used in the auxiliary telescope. The theory of operation is presented and then applied to a few cases for which the actual position of the polarizer is estimated, and the improvement of the volume depolarization ratio in the molecular region is quantified. In comparison to the method used before, i.e., without correction, the agreement between the volume depolarization ratio with correction and the theoretical value in the molecular region is improved by a factor of 2–2.5.

## 1. Introduction

The type and origin of atmospheric aerosols can be studied with the measurements provided by multiwavelength lidars. Those measurements provide useful products, among them [1] the lidar ratio (that relates the retrieved aerosol extinction and backscattering at the used wavelengths); the ratio of lidar ratios at two different wavelengths; and the color ratio or the Ängstrom exponent [2], which compares the retrieved backscattering and extinction at different wavelengths as well.

The lidar depolarization technique has been used since the 1970s and provides useful additional information for atmospheric sciences, see e.g., [3] or [4]. In the field of aerosol characterization, the information associated with the depolarization has been widely used in combination with the optical products indicated in the previous paragraph (see, e.g., [5,6,7,8,9,10]). Moreover, the depolarization ratio is extremely useful in the retrieval of the atmospheric boundary layer (ABL) height since it allows us to discriminate between the aerosol within this layer and different aerosol types coupled to the ABL height based on aerosol data [11]. Wandinger et al. [12] showed how different types of aerosols and clouds can be identified by using combined data, including color and depolarization ratios. In this manner, the usual set of lidar products can be combined with depolarization information to improve the aerosol classification algorithms (see [10,12,13]).

Beyond aerosol typing, useful information about the microphysical properties of the atmospheric aerosols is provided by depolarization measurement techniques. The main interest comes from the relation between the particle shape and the depolarization, improving the detection of non-spherical particles (see, e.g., [14,15,16,17]).

We have presented recently [18] the implementation of a new architecture for a depolarization lidar. It is based on a two-telescope arrangement: the main telescope collects (among other returns) the elastically backscattered light at 532 nm, without any polarization discrimination; the auxiliary telescope collects only the depolarized component of the backscattered light at the same wavelength. With this system, the volume depolarization ratio can be calculated by comparing the outputs of the two channels, taking into account a system calibration profile that includes information of the different responses of the channels [19]. The correct retrieval of the volume depolarization ratio is strongly dependent on the position of a polarization analyzer in the auxiliary channel, which must be oriented exactly at 90° from the transmitted linear polarization.

The purpose of this paper is to present a method that permits, from the outputs of the system calibration process, an estimation of the actual orientation of the polarization analyzer and to use this information to improve the calculation of the volume depolarization ratio.

The estimation of the actual orientation of the polarization analyzer is obtained by comparing the ratio of the signal of the depolarization channel over that of the total power channel at the two calibration positions (+45° and −45° from the nominal position) proposed by [19], at a distance from the lidar system where the atmosphere is assumed free of aerosols.

The estimation of the volume depolarization ratio [18], which is obtained from the comparison of the depolarization and total power channel outputs, can be corrected by considering that some amount of co-polar backscattered power is being detected because of the non-ideal position of the polarizer.

## 2. Proposed Method for Estimating the System Calibration and the Atmospheric Volume Depolarization Ratio

The lidar system measures along the vertical axis, so in the forthcoming the distance *R* from the lidar represents the height above the system.

The voltage signal obtained at a total power channel detector output can be written as

(1) $$S_{T}\left(R\right)=V_{T}\left(R\right)\cdot P_{T}\left(R\right)$$

where $V_{T}\left(R\right)$ is the elastic total power channel responsivity at distance *R*, including the effect of the partial overlap [20], the fiber bundle transmission, the losses in the wavelength separation unit and the detector responsivity.

$P_{T}\left(R\right)$ is the backscattered light power collected by the main telescope at the working wavelength, $P_{T}\left(R\right)=P_{∥}\left(R\right)+P_{⊥}\left(R\right)$, where $P_{∥}\left(R\right)$ and $P_{⊥}\left(R\right)$ are the co-polar and cross-polar received powers.

The voltage signal obtained at the depolarization (auxiliary) channel detector output can be written as

(2) $$S_{Dep}\left(\varphi ,R\right)=V_{Dep}\left(R\right)\cdot P_{\varphi }\left(R\right)$$

where $V_{Dep}\left(R\right)$ is the depolarization channel responsivity at distance *R*, including the effect of the partial overlap. In the case of a two-telescope system, $V_{Dep}\left(R\right)$ is different from $V_{T}\left(R\right)$, given the different positions and optical properties of the telescopes and also the—in general—different responsivities of the photo-receivers. $P_{\varphi }\left(R\right)$ is the fraction of the depolarized backscattered light at distance *R*, detected when the polarization analyzer is rotated by an angle φ with respect to the plane of polarization of the transmitted laser pulses. $P_{\varphi }\left(R\right)$ will have contributions from both the co-polar and cross-polar backscattered light, depending on φ:

(3) $$P_{\varphi }\left(R\right)=P_{\left|\right|}\left(R\right)\cos^{2}\varphi +P_{⊥}\left(R\right)\sin^{2}\varphi .$$

We will define the depolarization channel system function as

(4) $$V^{*}\left(R\right)=\frac{V_{Dep}\left(R\right)}{V_{T}\left(R\right)}.$$

While it is extremely difficult to estimate both $V_{Dep}\left(R\right)$ and $V_{T}\left(R\right)$, it is possible to determine $V^{*}\left(R\right)$ by means of a calibration process which compares the output signals of the total power and the depolarization channel.

Let us define the observable $\delta^{*}$ as

(5) $$\delta^{*}\left(\varphi ,R\right)=\frac{S_{Dep}\left(\varphi ,R\right)}{S_{T}\left(R\right)}=\frac{V_{Dep}\left(R\right)\cdot \left[P_{\left|\right|}\left(R\right)\cos^{2}\varphi +P_{⊥}\left(R\right)\sin^{2}\varphi \right]}{V_{T}\left(R\right)\cdot \left[P_{\left|\right|}\left(R\right)+P_{⊥}\left(R\right)\right]}.$$

Now let us define the linear volume depolarization produced by the atmosphere [19]:

(6) $$\delta^{V}\left(R\right)=\frac{P_{⊥}\left(R\right)}{P_{∥}\left(R\right)}$$

where $P_{⊥}\left(R\right)$ is equal to $P_{\varphi }\left(R\right)$ when φ=90°, containing only depolarized backscattered light. This is the first atmospheric depolarization parameter that can be measured and this paper is devoted to its correct estimation.

We can relate the observable $\delta^{*}\left(\varphi ,R\right)$ and the linear volume depolarization ratio $\delta^{V}\left(R\right)$ by dividing the numerator and denominator in Equation (5) by $P_{∥}\left(R\right)$:

(7) $$\delta^{*}\left(\varphi ,R\right)=V^{*}\left(R\right)\cdot \frac{\cos^{2}\varphi +\delta^{V}\left(R\right)\cdot \sin^{2}\varphi }{1+\delta^{V}\left(R\right)}.$$

In order to estimate $V^{*}\left(R\right)$, we will perform two measurements with two different values of φ separated by 90° with respect to a nominal $\varphi_{0}$ position:

(8) $$\begin{array}{l} \varphi_{-}=\varphi_{0}-45{}^{\circ}\\ \varphi_{+}=\varphi_{0}+45{}^{\circ}. \end{array}$$

Then,

(9) $$\begin{array}{l} \delta^{*}\left(\varphi_{-},R\right)=V^{*}\left(R\right)\cdot \frac{\cos^{2}\varphi_{-}+\delta^{V}\left(R\right)\cdot \sin^{2}\varphi_{-}}{1+\delta^{V}\left(R\right)}\\ \delta^{*}\left(\varphi_{+},R\right)=V^{*}\left(R\right)\cdot \frac{\sin^{2}\varphi_{-}+\delta^{V}\left(R\right)\cdot \cos^{2}\varphi_{-}}{1+\delta^{V}\left(R\right)}. \end{array}$$

Then we can obtain, independently of the specific value of $\varphi_{0}$,

(10) $$V^{*}\left(R\right)=\delta^{*}\left(\varphi_{-},R\right)+\delta^{*}\left(\varphi_{+},R\right).$$

When performing depolarization measurements, we will orientate the polarization analyzer at a $\varphi =\varphi_{0}$ position, which theoretically should be 90°. From Equation (7), we can obtain the volume depolarization by measuring the observable $\delta^{*}\left(90{}^{\circ},R\right)$ and calculating

(11) $$\delta^{V}\left(R\right)=\frac{\delta^{*}\left(90{}^{\circ},R\right)}{V^{*}\left(R\right)-\delta^{*}\left(90{}^{\circ},R\right)}.$$

This is the most common procedure to measure the volume depolarization ratio, as it is found in most references (see again [19]). Nevertheless, it relies on the exact value of φ=90° to avoid the crosstalk of the co-polar backscattered signal $P_{∥}\left(R\right)$ in the depolarization channel.

While it is relatively easy to ensure, by means of a simple mechanical setup, that the calibration of our depolarization channel is performed at two different positions for which the difference $\varphi_{+}-\varphi_{-}=90{}^{\circ}$ is established with errors below ±0.1°, the exact $\varphi_{0}$ position is prone to larger errors, as it can be affected by some mechanical error or by some instability of the polarization of the transmitted laser pulse.

To estimate the actual value of $\varphi_{0}$, we can subtract the two equations in (9):

(12) $$\begin{array}{ll} \delta^{*}\left(\varphi_{-},R\right)-\delta^{*}\left(\varphi_{+},R\right) & =V^{*}\left(R\right)\cdot \frac{1-\delta^{V}\left(R\right)}{1+\delta^{V}\left(R\right)}\cdot \cos2\varphi_{-}=\\ & =V^{*}\left(R\right)\cdot \frac{1-\delta^{V}\left(R\right)}{1+\delta^{V}\left(R\right)}\cdot \sin2\varphi_{0}=\\ & =\left[\delta^{*}\left(\varphi_{-},R\right)+\delta^{*}\left(\varphi_{+},R\right)\right]\cdot \frac{1-\delta^{V}\left(R\right)}{1+\delta^{V}\left(R\right)}\cdot \sin2\varphi_{0} \end{array}.$$

Behrendt and Nakamura [21] computed $\delta^{V}\left(R\right)$ in detail for the so-called molecular atmosphere, i.e., the depolarization of light backscattered by molecules only. This value, denoted $\delta_{m}^{V}$, depends, basically, on the spectral width of the interference filter used in the depolarization channel. A calibration of the actual value of $\varphi_{0}$ can be performed by selecting some part of our signal which comes from a part of the atmosphere free of aerosols at distance $R_{mol}$, so that $\delta^{V}=\delta_{m}^{V}:$

(13) $$\sin2\varphi_{0}=\frac{1+\delta_{m}^{V}}{1-\delta_{m}^{V}}\cdot \frac{\delta^{*}\left(\varphi_{-},R_{mol}\right)-\delta^{*}\left(\varphi_{+},R_{mol}\right)}{\delta^{*}\left(\varphi_{-},R_{mol}\right)+\delta^{*}\left(\varphi_{+},R_{mol}\right)}\approx \frac{\delta^{*}\left(\varphi_{-},R_{mol}\right)-\delta^{*}\left(\varphi_{+},R_{mol}\right)}{\delta^{*}\left(\varphi_{-},R_{mol}\right)+\delta^{*}\left(\varphi_{+},R_{mol}\right)}$$

where $\delta_{m}^{V}\ll 1$ has been used (the value calculated by Behrendt and Nakamura [21] according to the parameters of our system is 3.8 × 10^−3^). The values of $\delta^{*}\left(\varphi_{-},R_{mol}\right)$ and $\delta^{*}\left(\varphi_{+},R_{mol}\right)$ are expected to be very small, so we will use mainly night-time measurements, averaged over 150 min, to estimate $\varphi_{0}$.

The knowledge of the actual value of $\varphi_{0}$ allows a better estimation of the volume depolarization ratio in any measurement by solving for $\delta^{V}\left(R\right)$ in Equation (7):

(14) $$\delta^{V}\left(R\right)=\frac{\delta^{*}\left(\varphi_{0},R\right)-V^{*}\left(R\right)\cdot \cos^{2}\varphi_{0}}{V^{*}\left(R\right)\cdot \sin^{2}\varphi_{0}-\delta^{*}\left(\varphi_{0},R\right)}$$

which is equal to Equation (11) for $\varphi_{0}=90{}^{\circ}$.

## 3. Calibration and Estimation of the Polarizer Angle

CommSensLab has developed a multiwavelength Raman lidar (a complete description is available in [22]), which has been recently upgraded with an auxiliary depolarization channel [18].

The transmitter is a Quantel-laser^®^ Brilliant^®^ laser (Quantel, Les Ulis, France), equipped with a second and third harmonic generator, with 4 ns pulses with approximate energies of 130 mJ at 1064 nm and 532 nm and 40 mJ at 355 nm. The 6-channel main receiver (sensitive to the total collected power) unit is based on a 356 mm diameter telescope (C14-A XLT, CELESTRON^®^, Torrance, CA, USA) which collects the light backscattered by the atmosphere and couples it (through a field lens) to a 3 mm diameter multimode fiber bundle (manufactured by CeramOptec^®^, custom-made, CeramOptec, Bonn, Germany), with an estimated overall field of view of 1.2 mrad. The fiber bundle delivers the light to a wavelength separation unit, which splits the light to the different channels: three elastic (1064 nm, 532 nm and 355 nm) and three Raman channels (607 nm and 387 nm for nitrogen excited at 532 nm and 355 nm, respectively, and 407 nm for water vapor excited at 355 nm).

The aerosol depolarization auxiliary channel [18] uses a separate telescope (a 70 mm aperture, 300 mm focal distance TAIR-3S telephoto lens, BelOMO, Minsk, Belarus). The rest of the optical setup includes a 1-mm field of view (FOV) stop iris (which provides an approximate FOV of 3.33 mrad), a polarization analyzer, an eye-piece lens, and an interference filter (Barr Associates, Inc., Westford, MA, USA), centered at 532 nm with a spectral width of 0.5 nm. The polarization analysis is carried out by means of a linear polarizer mounted close to the iris that can be rotated in a controlled manner by means of a goniometric mount.

Neither the main nor the auxiliary telescopes are co-axial with the laser transmitter, nor are their fields of view the same, which leads to different overlap functions for the main and auxiliary receiving channels, especially at short distances (see, e.g., [20,23,24]).

**Figure 1:**
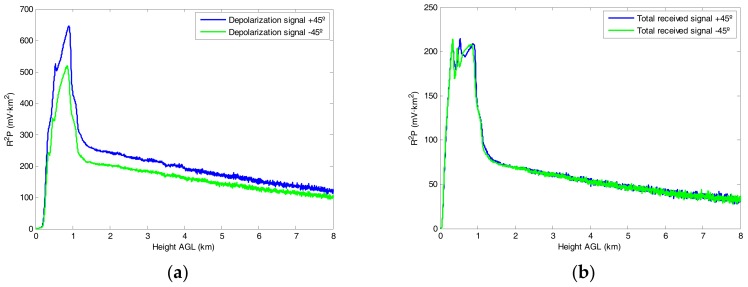
Depolarization signals (**a**) and total received signals (**b**) used in the calibration procedure.

To illustrate the method proposed in Section 2, we present the results of a calibration performed on 15 March 2017. We perform both the +45° and −45° calibrations during two consecutive 15 min measurements. Figure 1 shows the signals collected by the auxiliary (depolarization) telescope during the two calibrations in the left panel, and the signals collected by the main (total power) telescope in the right panel. It is to be noted that, while the total power signal collected has similar values for the two calibrations, the depolarization calibration signal is stronger in the case of the +45° position than for the −45° position. We attribute this difference to an error in the setting of the nominal position of the polarizer of the depolarization channel, as explained in Section 2.

**Figure 2:**
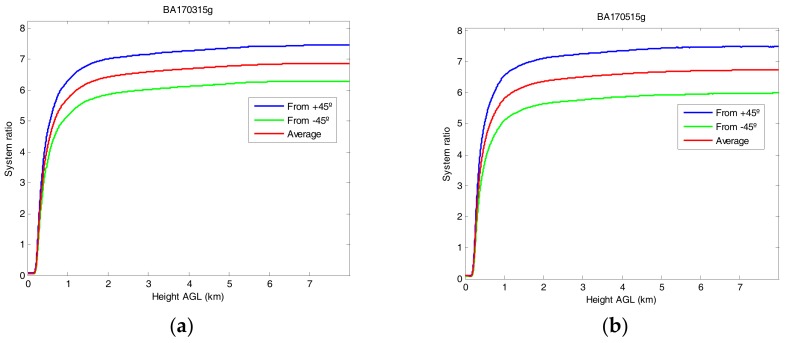
Profiles of $2\delta^{*}\left(\varphi_{0}-45{}^{\circ},R\right)$ (green), $2\delta^{*}\left(\varphi_{0}+45{}^{\circ},R\right)$ (blue) and $V^{*}\left(R\right)$ (red) for calibrations performed on (**a**) 15 March 2017 and (**b**) 15 May 2017.

Figure 2 shows, for two different settings of the alignment of the telescopes, the profiles of $\delta^{*}\left(\varphi_{0}-45{}^{\circ},R\right)$ and $\delta^{*}\left(\varphi_{0}+45{}^{\circ},R\right)$, multiplied by 2 to emphasize the differences of the two profiles and compare them with their sum, $V^{*}\left(R\right)$, which will be used according to Equation (10) as the depolarization system function. In both cases the system ratio profile shows that the full overlap of the depolarization auxiliary telescope was obtained farther than that of the main, total power receiver channel. The signal obtained in the auxiliary depolarization channel is around 6.5 times stronger than in the main receiver, but its full overlap is not reached until approximately 7 km.

It must be stressed that the system ratio profile is considered to be constant from 6 km to 8 km on for conceptual and practical reasons: on the one hand, once that full overlap is reached in both channels, this ratio should be constant; on the other hand, the signal-to-noise ratio degrades with altitude.

As it was indicated in Equation (13), we use the values obtained in the molecular zone to estimate the actual value of the polarizer position with respect to the transmitted beam polarization. The estimated polarizer position angle is reported in Table 1 for the two calibrations shown in Figure 2 and six other calibrations. For the two calibrations shown in Figure 2, we find $\varphi_{0}=92.5{}^{\circ}\pm 0.1{}^{\circ}$ for Figure 2a and $\varphi_{0}=93.2{}^{\circ}\pm 0.1{}^{\circ}$ for Figure 2b. Over the whole set of values of $\varphi_{0}$ reported in Table 1, we find a range of deviations from the theoretical 90° value comprised between −2.5° and +4.2°.

## 4. Effect of Correction on Estimation of the Volume Depolarization

**Figure 3:**
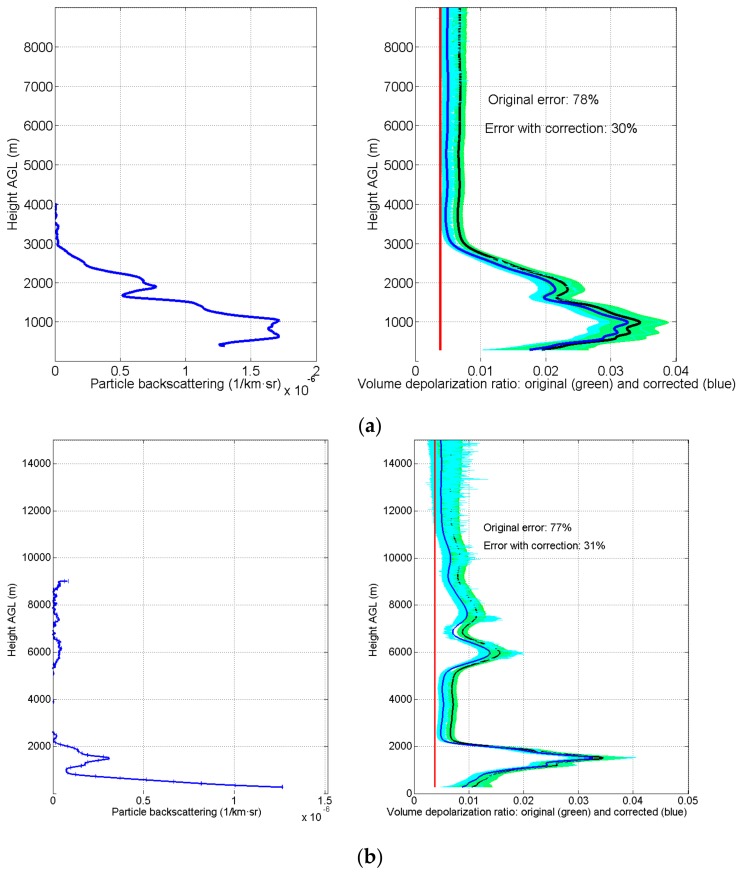

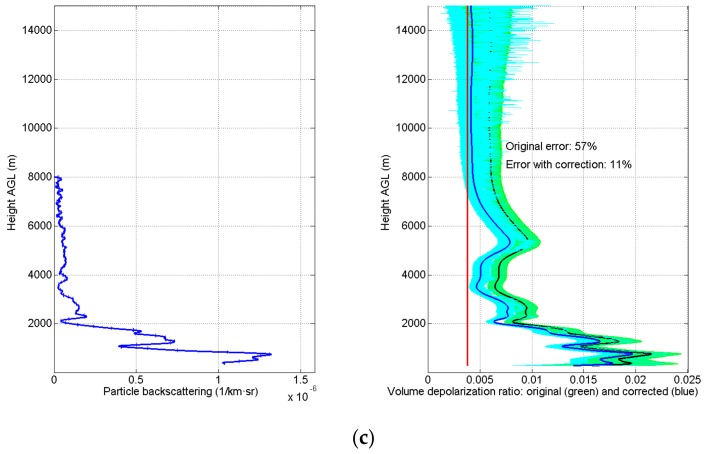
Effect of correction on estimation of the volume depolarization for three different night-time measurements ((**a**) 19 March 2017, (**b**) 28 March 2017, (**c**) 13 April 2017) performed in March and April 2017. The left panels show the particle backscattering retrieved using the Raman algorithm.

The three panels of Figure 3 present the result of the volume depolarization ratio of three different night-time measurements performed during March and April 2017 with the calibration performed on 15 March 2017 and presented in Section 3. We have used night-time measurements (performed over 150 min intervals) to avoid the effect of background noise. In each panel, the retrieved particle backscattering (by means of the Raman algorithm [25]) is presented for comparison purposes. The presence of some slightly negative values of the retrieved particle backscattering coefficient is mainly due to small systematic errors in a zone of nearly molecular atmosphere; nevertheless, the presented volume depolarization profiles do not depend on this particle backscattering profile.

The volume depolarization ratio value has been computed according to Equation (11) and compared with the corrected value given by Equation (14) and the actual position of the polarizer of $\varphi_{0}=92.5{}^{\circ}$ calculated previously for the calibration performed on 15 March 2017. The volume depolarization ratio is computed up to height values where it is assumed that the aerosol content of the atmosphere is negligible and the only contribution to the depolarization is that due to the atmospheric molecules. We have compared the value of the volume depolarization in the part of the atmosphere assumed free of aerosols obtained from our measurements with the value $\delta_{m}^{V}=3.8\times 10^{-3}$ calculated by Behrendt and Nakamura [21] and corresponding to a spectral bandwidth of 0.5 nm around 532 nm. In the measurement presented in Figure 3a, it is assumed that the atmosphere is free of aerosols from 3.5 km to 8 km, while in Figure 3b this is assumed from 11 km to 14.5 km and in Figure 3c from 7 km to 14.5 km. The averaged relative error with respect to the theoretical value of $\delta_{m}^{V}$ is presented for both calculations of the volume depolarization ratio, with the finding that it is significantly smaller for the corrected value of $\delta^{V}\left(R\right)$ provided by Equation (14); more specifically, there are reductions from 78% to 30% for Figure 3a, from 77% to 31% for Figure 3b, and from 57% to 11% for Figure 3c. Thus, in all three cases, the relative error reduction is better than 50%.

## 5. Conclusions

We have proposed a new method for computing the volume depolarization ratio for lidar systems which use a separate telescope for detecting the light depolarized by the atmosphere. It takes into account the non-ideal positioning of the polarizer used in the auxiliary channel (which can be estimated during the calibration process) to perform a correction of the calculated value. The effect of this correction can be evaluated when the volume depolarization ratio obtained in a region of the atmosphere which can be assumed free of aerosols is compared with the theoretical value. The observed effect is a significant reduction (of at least 50%) of the relative deviation from the theoretical value, according to the experimental results presented. 

## Figures and Tables

**Table 1:** Polarizer estimated positions for different calibrations performed during 2017 and 2018.

Calibration Date	Molecular Atmosphere Range Considered (m)	Actual Angle Position, $\varphi_{0}$(°)
9 January 2017	7500–8000	90.7 ± 0.1
15 March 2017	7500–8000	92.5 ± 0.1
15 May 2017	7500–8000	93.2 ± 0.1
1 June 2017	7500–8000	93.3 ± 0.1
24 October 2017	5500–6000	87.5 ± 0.1
27 November 2017	6000–6500	88.1 ± 0.1
21 February 2018	4500–5000	90.4 ± 0.1
9 April 2018	5500–6000	94.2 ± 0.1

## References

[1] Müller D., Ansmann A., Mattis I., Tesche M., Wandinger U., Althausen D., Pisani G. (2007). Aerosol-type-dependent lidar ratios observed with Raman lidar. J. Geophys. Res..

[2] Angstrom B.A., Eppley T. (1964). The parameters of atmospheric turbidity. Tellus.

[3] Schotland R.M., Sassen K., Stone R. (1971). Observations by lidar of linear depolarization ratios for hydrometeors. J. Appl. Meteorol..

[4] Pal S.R., Carswell A.I. (1973). Polarization properties of lidar backscattering from clouds. Appl. Opt..

[5] Winker D.M., Osborn M.T. (1992). Airborne lidar observations of the Pinatubo volcanic plume. Geophys. Res. Lett..

[6] Murayama T., Müller D., Wada K., Shimizu A., Sekiguchi M., Tsukamoto T. (2004). Characterization of Asian dust and Siberian smoke with multi-wavelength Raman lidar over Tokyo, Japan in spring 2003. Geophys. Res. Lett..

[7] Tafuro A.M., Barnaba F., De Tomasi F., Perrone M.R., Gobbi G.P. (2006). Saharan dust particle properties over the central Mediterranean. Atmos. Res..

[8] Tesche M., Ansmann A., Muller D., Althausen D., Mattis I., Heese B., Freudenthaler V., Wiegner M., Esselborn M., Pisani G. (2009). Vertical profiling of Saharan dust with Raman lidars and airborne HSRL in southern Morocco during SAMUM. Tellus B.

[9] Groß S., Gasteiger J., Freudenthaler V., Wiegner M., Geiß A., Schladitz A., Toledano C., Kandler K., Tesche M., Ansmann A. (2011). Characterization of the planetary boundary layer during SAMUM-2 by means of lidar measurements. Tellus B Chem. Phys. Meteorol..

[10] Groß S., Tesche M., Freudenthaler V., Toledano C., Wiegner M., Ansmann A., Althausen D., Seefeldner M. (2011). Characterization of Saharan dust, marine aerosols and mixtures of biomass-burning aerosols and dust by means of multi-wavelength depolarization and Raman lidar measurements during SAMUM 2. Tellus B Chem. Phys. Meteorol..

[11] Bravo-Aranda J.A., de Arruda Moreira G., Moreira A., Navas-Guzmán F., Granados-Muñoz M.J., Guerrero-Rascado J.L., Pozo-Vázquez D., Arbizu-Barrena C., José F., Reyes O. (2017). A new methodology for PBL height estimations based on lidar depolarization measurements: analysis and comparison against MWR and WRF model-based results. Atmos. Chem. Phys..

[12] Wandinger U., Ansmann A., Mattis I., Müller D., Pappalardo G. CALIPSO and beyong: Long-term ground-based support of space-borne aerosols and cloud lidar missions. Proceedings of the 24th International Laser Radar Conference.

[13] Burton S.P., Ferrare R.A., Hostetler C.A., Hair J.W., Rogers R.R., Obland M.D., Butler C.F., Cook A.L., Harper D.B., Froyd K.D. (2012). Aerosol classification using airborne High Spectral Resolution Lidar measurements-methodology and examples. Atmos. Meas. Tech..

[14] Burton S.P., Hair J.W., Kahnert M., Ferrare R.A., Hostetler C.A., Cook A.L., Harper D.B., Berkoff T.A., Seaman S.T., Collins J.E. (2015). Observations of the spectral dependence of linear particle depolarization ratio of aerosols using NASA Langley airborne High Spectral Resolution Lidar. Atmos. Chem. Phys..

[15] Olmo F.J., Quirantes A., Lara V., Lyamani H., Alados-Arboledas L. (2008). Aerosol optical properties assessed by an inversion method using the solar principal plane for non-spherical particles. J. Quant. Spectrosc. Radiat. Transf..

[16] Veselovskii I., Goloub P., Podvin T., Bovchaliuk V., Derimian Y., Augustin P., Fourmentin M., Tanre D., Korenskiy M., Whiteman D.N. (2016). Retrieval of optical and physical properties of African dust from multiwavelength Raman lidar measurements during the SHADOW campaign in Senegal. Atmos. Chem. Phys..

[17] Müller D., Veselovskii I., Kolgotin A., Tesche M., Ansmann A., Dubovik O. (2013). Vertical profiles of pure dust and mixed smoke–dust plumes inferred from inversion of multiwavelength Raman/polarization lidar data and comparison to AERONET retrievals and in situ observations. Appl. Opt..

[18] Rodríguez-Gómez A., Sicard M., Granados-Muñoz M.J., Ben Chahed E., Muñoz-Porcar C., Barragán R., Comerón A., Rocadenbosch F., Vidal E. (2017). An architecture providing depolarization ratio capability for a multi-wavelength raman lidar: Implementation and first measurements. Sensors.

[19] Freudenthaler V., Esselborn M., Wiegner M., Heese B., Tesche M., Ansmann A., Müller D., Althausen D., Wirth M., Fix A. (2009). Depolarization ratio profiling at several wavelengths in pure Saharan dust during SAMUM 2006. Tellus B Chem. Phys. Meteorol..

[20] Wandinger U., Weitkamp C. (2005). Introduction to Lidar. Lidar.

[21] Behrendt A., Nakamura T. (2002). Calculation of the calibration constant of polarization lidar and its dependency on atmospheric temperature. Opt. Express.

[22] Kumar D., Rocadenbosch F., Sicard M., Comeron A., Lange D., Muñoz C., Tomás S., Gregorio E. Six-channel polychromator design and implementation for the UPC elastic/Raman LIDAR. Proceedings of the Lidar Technologies, Techniques, and Measurements for Atmospheric Remote Sensing VII.

[23] Halldórsson T., Langerholc J. (1978). Geometrical form factors for the lidar function. Appl. Opt..

[24] Comeron A., Sicard M., Kumar D., Rocadenbosch F. (2011). Use of a field lens for improving the overlap function of a lidar system employing an optical fiber in the receiver assembly. Appl. Opt..

[25] Ansmann A., Riebesell M., Weitkamp C. (1990). Measurement of atmospheric aerosol extinction profiles with a Raman lidar. Opt. Lett..

